# Examining the automaticity and symmetry of sound–shape correspondences

**DOI:** 10.3389/fpsyg.2023.1172946

**Published:** 2023-06-05

**Authors:** Yi-Chuan Chen, Pi-Chun Huang

**Affiliations:** ^1^Department of Medicine, MacKay Medical College, New Taipei City, Taiwan; ^2^Department of Psychology, National Cheng Kung University, Tainan, Taiwan

**Keywords:** crossmodal correspondences, implicit association test, speeded classification task, automatic processing, bidirectional association

## Abstract

**Introduction:**

A classic example of sound–shape correspondences is the mapping of the vowel /i/ with angular patterns and the vowel /u/ with rounded patterns. Such crossmodal correspondences have been reliably reported when tested in explicit matching tasks. Nevertheless, it remains unclear whether such sound–shape correspondences automatically occur and bidirectionally modulate people’s perception. We address this question by adopting the explicit matching task and two implicit tasks.

**Methods:**

In Experiment 1, we examined the sound–shape correspondences using the implicit association test (IAT), in which the sounds and shapes were both task-relevant, followed by an explicit matching task. In Experiments 2 and 3, we adopted the speeded classification task; when the target was a sound (or shape), a task-irrelevant shape (or sound) that was congruent or incongruent to the target was simultaneously presented. In addition, the participants performed the explicit matching task either before or after the speeded classification task.

**Results and Discussion:**

The congruency effect was more pronounced in the IAT than in the speeded classification task; in addition, a bin analysis of RTs revealed that the congruency effect took time to develop. These findings suggest that the sound–shape correspondences were not completely automatic. The magnitude and onset of visual and auditory congruency effects were comparable, suggesting that the crossmodal modulations were symmetrical. Taken together, the sound–shape correspondences appeared not to be completely automatic, but their modulation was bidirectionally symmetrical once it occurred.

## 1. Introduction

**Figure 1:**
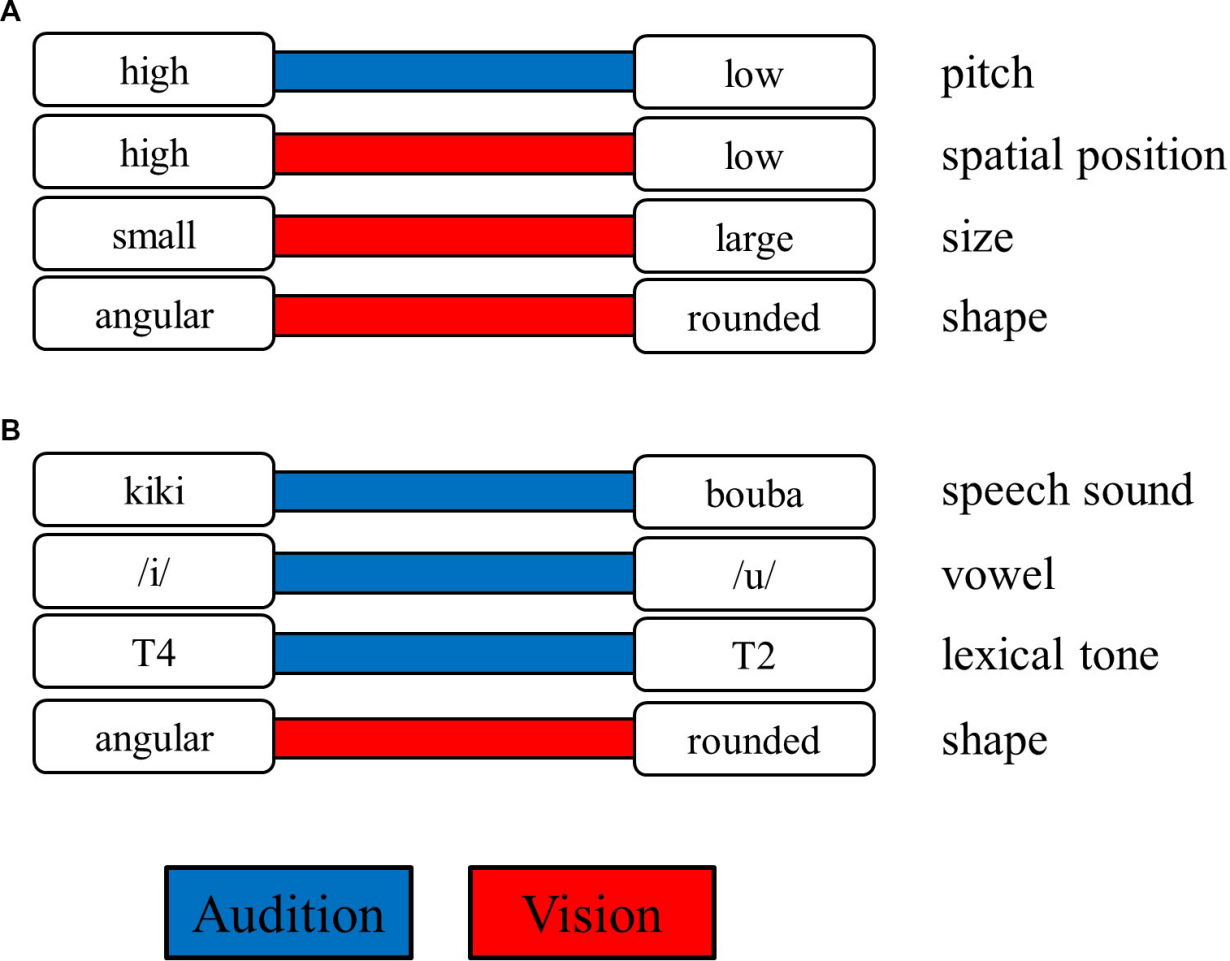
Examples of crossmodal correspondences. The contrasting features (in the white rectangles, such as the sound of high vs. low pitch) are connected by a colored bar either in the auditory modality (in blue) or in the visual modality (in red). The features aligned in the left and right columns correspond to each other (e.g., high pitch and high spatial position). **(A)** Illustrates the correspondences associated with pitch, and **(B)** illustrates the correspondence associated with speech sound. T2 and T4 represent lexical tone 2 (consisting of a rising pitch contour) and lexical tone 4 (consisting of a falling pitch contour) in Mandarin (see Figure 2 for details).

Multisensory signals originating from the same object or event are often correlated rather than arbitrary in some properties, and such crossmodal correspondences provide critical cues for human brains to integrate these signals (e.g., Spence, 2011; Chen and Spence, 2017). For example, a bird’s chirping is high-pitched and often associated with its small body, pointy beak, and high elevation on the tree; in contrast, a cow’s mooing is low-pitched and often associated with its huge and rounded body and low position on the ground (See Figure 1A; Marks, 1987; Tsur, 2006; Lowe and Haws, 2017). In addition to simple features, crossmodal correspondences between complex stimuli have also been well documented, such that the meaningless speech sounds “kiki” and “bouba” are often matched with angular and rounded shapes, respectively, rather than the reverse (see Figure 1B; Ramachandran and Hubbard, 2001; see Köhler, 1929, 1947; Holland and Wertheimer, 1964 for early studies). In the current study, we aimed to investigate whether crossmodal correspondences, specifically the sound–shape correspondences, can occur automatically and symmetrically in influencing the auditory and visual information processing (see Spence and Deroy, 2013, on this issue).

Over the years, researchers have commonly used explicit tasks to explore new crossmodal correspondences. Köhler (1929), for example, explicitly asked participants to match the nonwords “takete” and “baluma” to two line drawings—one with sharp angles and the other with smooth curves. In another study, Sapir (1929) asked participants whether the vowel /a/ symbolized the concept of “large” compared to the vowel /i/. In such *explicit matching tasks*, the participants often only made a single judgment. When a consensual match among this group of participants was reached, the tested correspondences could be confirmed (e.g., Rogers and Ross, 1975; Bremner et al., 2013). Other explicit methods can directly quantify the strength of crossmodal correspondences, such as presenting participants a target stimulus (e.g., sweet liquid) and asking them to rate or adjust a feature along a bipolar dimension in another sensory modality (e.g., a sound’s pitch) that best matches the target (e.g., Stevens and Marks, 1965; Crisinel and Spence, 2010; Ngo et al., 2013).

These explicit tasks provide straightforward methods and fruitful outcomes in understanding crossmodal correspondences (e.g., see Spence and Sathian, 2020, for a review). However, the explicit methods have at least three limitations. First, the explicit tasks rely on the participants’ introspective ability (see Parise and Spence, 2012; Westbury et al., 2018). Even though Chen et al. (2019) demonstrated that most of their participants who were healthy adults have good introspection when tested with sound–shape correspondences, caution is advised when one explores crossmodal correspondences in people of other ages (such as young children) or special groups (such as those with autistic spectrum disorders, see Spence, 2022, for a review). Second, given that participants are generally being forced to respond in the explicit tasks, the observed associations may be a product of the decisional/response rather than perceptual/cognitive processing. Third, explicit tasks depend on conscious judgments; therefore, the responses are susceptible to individual tendencies, preferences, and intentions. In sum, the responses or judgments in the explicit tasks are prone to be generated from decisional, voluntary, and controlled processing.

To investigate whether crossmodal correspondences occur at the perceptual/cognitive level rather than the decisional/response level, researchers have adopted more sophisticated psychophysical paradigms. One of these tasks is the implicit association test (IAT), which has often been used to measure the strength of associations between two pairs of items or concepts (e.g., the associations between flower–pleasant and insect–unpleasant, Greenwald et al., 1998). In the IAT, a single stimulus is presented in each trial and participants have to categorize the stimulus rapidly and accurately by pressing one of two predesignated response keys (often using their left and right hands). Within an experimental block of trials, the mappings between the stimuli and response keys were either congruent (e.g., “flower” and “pleasant” correspond to the same response key) or incongruent (e.g., “flower” and “unpleasant” correspond to the same response key). It is then assumed that if participants intrinsically associated the items as expected, the response time (RT) would be shorter and/or the accuracy would be higher in the congruent than in the incongruent condition (called the congruency effect). Furthermore, the magnitude of the congruency effect can be a quantitative index of the strength of associations. Taken together, in the IAT, participants judged each item on its merits rather than the associations between items. Therefore, in the IAT, crossmodal correspondences are implicitly encoded in the stimulus–response mappings rather than serving as explicit task goals.

Parise and Spence (2012) utilized the IAT to investigate the core level of processing on five different pairs of crossmodal correspondences. They reported reliable and similar magnitudes of congruency effect regardless of the stimuli’s complexity (e.g., pitch–size correspondences rely on simpler stimuli than sound–shape correspondences). By conducting bin analysis of RTs (i.e., the RTs were ordered from the fastest to slowest and then evenly divided into five time bins; see Data Analysis section), Parise and Spence demonstrated that the congruency effect appeared in the initial time bin. Therefore, the rapid onset of the congruency effect suggested that crossmodal correspondences can even occur at the early stage of information processing. Parise and Spence further analyzed the congruency effects in the trials containing a visual or auditory target and demonstrated that their magnitudes were similar, suggesting that the influence in terms of crossmodal correspondences from one to the other modality was bidirectional and symmetrical.

The IAT provides an indirect measure to inspect the role of crossmodal correspondences at the perceptual/cognitive level of information processing. However, the congruency effect observed in the IAT could not unequivocally indicate an *implicit* and *automatic* association. For example, because the congruent and incongruent conditions were tested in different blocks, participants may be aware of the relationships between the stimuli; therefore, the congruency effect reflects implicit and explicit associations or attitudes (see Schimmack, 2021; though see Kurdi et al., 2021). It is also plausible that given the repeated presentations of the stimuli and the well-practiced stimulus–response mappings, certain associations may be learned during the course of the experiment (Hussey and De Houwer, 2018). Therefore, to test whether crossmodal correspondences can occur automatically, adopting other psychophysical paradigms is necessary.

Another implicit task that has been used in the research on crossmodal correspondences is the *speeded classification task* (e.g., Marks, 1987; Melara and O'Brien, 1987; Martino and Marks, 1999; Gallace and Spence, 2006; Evans and Treisman, 2010). In this paradigm, participants quickly respond to the target presented in one modality while ignoring the distractor in the other modality, which is task-irrelevant and lacks an assigned response. The target and distractor are either crossmodally correspondent (i.e., congruent) or not (i.e., incongruent), and the two conditions are intermixed randomly within a block. If the RT is shorter and/or accuracy is higher in the congruent than in the incongruent condition, a typical congruency effect is observed, which suggests that the distractor is processed and facilitates or interferes with the target processing.

Gallace and Spence (2006), for example, asked participants to judge whether the target disc is larger or smaller than the preceding standard disc as rapidly and accurately as possible, and a task-irrelevant tone either in a higher pitch (4,500 Hz) or lower pitch (300 Hz), or else no tone, was simultaneously presented with the target disc. The participants’ RT was shorter when the target was paired with a congruent tone (e.g., a smaller disc and the higher-pitched tone) than when paired with an incongruent tone (e.g., a smaller disc and the lower-pitched tone), demonstrating that pitch–size correspondences had occurred. Evans and Treisman (2010) similarly utilized the speeded classification tasks to investigate the crossmodal correspondences’ bidirectional influences. In each trial, the researchers presented a tone (either a lower or higher pitch) and a visual disc (either large or small) and asked the participants to discriminate the pitch as either high or low while ignoring the visual disc (i.e., the auditory task) or to discriminate the disc as either large or small while ignoring the pitch (i.e., the visual task). Evans and Treisman found that the congruency effect was significant in the auditory and visual tasks; therefore, the modulation in terms of crossmodal correspondences was bidirectional.

In previous studies, we tested the sound–shape correspondences (e.g., the mapping of “kiki” and “bouba” to angular and rounded patterns) using explicit matching tasks (Chen et al., 2016, 2019, 2021; Chang et al., 2021; Shen et al., 2022). Researchers have also tried to use implicit tasks to investigate the level of processing at which sound–shape correspondences occur. For example, a reliable congruency effect has been reported in the IAT (Parise and Spence, 2012; Shang and Styles, 2023). In addition, the presentation of a congruent as compared to an incongruent sound enhanced the visibility of a masked visual pattern (as indicated by the lower contrast threshold, Hung et al., 2017). These results suggest that the sound–shape correspondences occurred at the perceptual/cognitive level rather than the decisional/response level. In the present study, we aimed to investigate the automaticity and symmetry of the sound–shape correspondences using two implicit tasks, the IAT and speeded classification tasks, and the explicit matching tasks.

According to early research comparing automatic and controlled processing, the former is suggested to be involuntary, capacity-unlimited, and pre-attentive (Schneider and Shiffrin, 1977). Moors and De Houwer (2006) proposed similar criteria for automatic processing—unintentional, efficient, and unconscious, and added the fourth criterion of speed; that is, an automatic process should occur quickly and influence the early stage of information processing. These four criteria are conceptually distinct, allowing them to be investigated individually. In addition, some of the criteria change in degrees rather than qualitatively. According to these propositions, the transition from automatic to controlled processing should occur gradually because there is no single dichotomous criterion (Moors and De Houwer, 2006). Following this gradual approach of automatic processing, we compared the congruency effect in the IAT and speeded classification tasks in terms of the aforementioned criteria.

In Experiments 1, 2, and 3, each participant performed the IAT, sound classification, or shape classification as the main task, respectively, either preceding or following the explicit matching tasks. Considering that RT was the main dependent variable in these implicit tasks, the presentation duration of visual and auditory stimuli should be equalized. Therefore, we utilized the vowels as the auditory stimuli: the vowels /i/ and /u/ are reliably matched to angular and rounded patterns, respectively (Sapir, 1929; Newman, 1933; Taylor and Taylor, 1962; Remez, 2003; Berlin, 2006; Maurer et al., 2006; Kovic et al., 2010; Lockwood and Dingemanse, 2015; Peiffer-Smadja and Cohen, 2019). The *automaticity* of the sound–shape correspondences was examined using the following tests in the implicit tasks: (1) According to the involuntariness (or unintentionality) criterion, the congruency effect should be similar in magnitude in the IAT and speeded classification tasks even though both modalities are task-relevant in the IAT whereas only the target modality was task-relevant in the speeded classification tasks. (2) According to the pre-attentiveness (or unconsciousness) criterion, the congruency effect should be significant irrespective of the order of the explicit matching and implicit tasks. This is because performing the explicit matching tasks would bring the concept of sound–shape correspondences to participants’ consciousness; however, an automatic process should occur irrespective of participants’ awareness of the correspondences. (3) According to the speed criterion, the congruency effect should emerge when the RT is short (e.g., in the first time bin in the bin analysis of RTs, see Parise and Spence, 2012). The *symmetry* of the sound–shape correspondences was examined using the following tests in the implicit tasks: (1) the magnitude of auditory and visual congruency effect should be similar, and (2) the auditory and visual congruency effect should emerge in similar time bins. These comparisons would provide detailed examinations of the automaticity and symmetry issues.

## 2. General methods

### 2.1. Participants

One hundred and twenty participants (24 participants in each experiment) took part in this study. Table 1 provides the participants’ demographic information. All of the participants had normal or corrected-to-normal vision and normal hearing by self-report. They were naïve to the purpose of the study and gave their informed consent before the experiment. All of the participants were students at National Cheng Kung University in Taiwan and native Mandarin Chinese speakers. They received monetary compensation for their participation. The study was conducted in accordance with the ethical standards of the Declaration of Helsinki and approved by the National Cheng Kung University research ethics committee for human behavioral sciences (NCKU HREC-E-110-460-2).

**Table 1:** Demographic information in three experiments.

Experiment	Main task	Explicit matching task (before/after main task)	Mean age (SD)	Gender (M/F)
1	Implicit association test (IAT)	After	20.4 (1.62)	(12/12)
2A	Sound classification	Before	21.4 (1.86)	(10/14)
2B		After	20.4 (1.57)	(12/12)
3A	Shape classification	Before	21.5 (2.55)	(12/12)
3B		After	20.9 (2.06)	(13/11)

To demonstrate the congruency effect of RT in the IAT and speeded classification tasks, we computed the *d-score*, a standardized difference between the RTs in the congruent and incongruent conditions. A d-score greater than zero indicates that a typical congruency effect was significant. An analysis using G*Power (version 3.1.9.4; Faul et al., 2007) for the one-sample *t*-test (one-tailed) suggested that when we test 24 participants with α = 0.05 and a power = 0.80, the effect size of the *t*-test would reach 0.52 (a medium effect). The sample (24 participants) was also larger than those in previous studies testing crossmodal correspondences using the IAT (*N* = 10, Parise and Spence, 2012) and speeded classification tasks (*N* = 10 or 15, Gallace and Spence, 2006). We were therefore confident that the number of participants provided adequate effect size to reach statistical significance.

### 2.2. Apparatus and stimuli

The experimental program was controlled by a computer compatible with the Psychophysics Toolbox (Brainard, 1997) in the MATLAB (The Mathworks, Matick, MA, USA) environment. The visual stimuli were presented on a liquid crystal display monitor (ASUS XG250) with a resolution of 1,920 × 1,080 pixels, running at 85 Hz. The viewing distance was 100 cm. The auditory stimuli were delivered by closed-ear headphones (Beyer dynamic DT770 Pro). The volume was set to 68 dB. The experiments were conducted in a soundproof, dim room.

**Figure 2:**
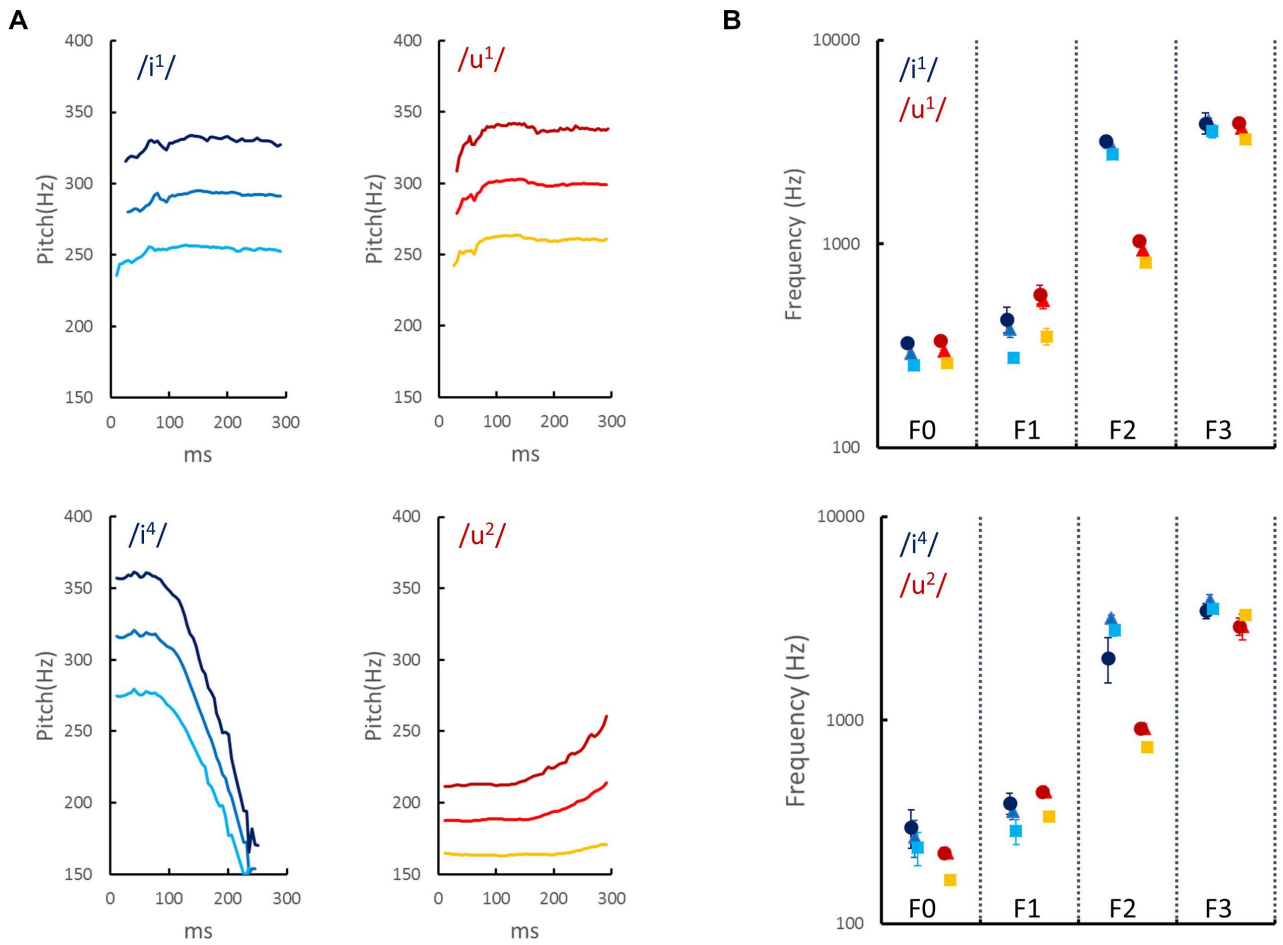
Acoustic properties of auditory stimuli used in the current study. **(A)** The temporal contours of the fundamental frequencies (F0s) of two vowels (/i/ and /u/) that were articulated in lexical tone 1 (/i1/ and /u1/ in the upper panels) or in lexical tones 4 and 2 (/i4/ and /u2/ in the lower panels). The bright, intermediate, and dark colors represent voices in the low, medium, and high frequencies, respectively. **(B)** The mean and standard deviation across time of the F0, the first formant (F1), the second formant (F2), and the third formant (F3). The acoustic properties were measured using Praat (Boersma, 2001). The frequencies of formants are associated with the articulation method: F1 increases with lower tongue position and greater jaw opening, and F2 is related to vowel frontness (e.g., the front vowel /i/ has a higher F2 than the back vowel /u/; Ladefoged and Disner, 2012).

The auditory stimuli were two pairs of tonal vowels to increase the variety of auditory stimuli. The first pair was the high front vowel /i/ and the high back vowel /u/, and both were articulated in Tone 1 (i.e., /i1/ and /u1/). The second pair consisted of the same vowels, and /i/ was articulated in Tone 4, and /u/ was articulated in Tone 2 (i.e., /i4/ and /u2/). Each pair of tonal vowels was presumably matched to angular and rounded patterns, respectively (see Figure 1B; Chang et al., 2021). These tonal vowels were produced by a female native speaker who teaches Mandarin Chinese pronunciation at the Chinese Language Center at National Cheng Kung University. These tonal vowels were recorded in a soundproof recording room using a Yeti microphone (Blue, CA, USA) and QuickTime Player (ver. 10.5, Apple Inc., CA, USA) at a 44.1-kHz sampling rate with 16-bit encoding. The audio files were then edited and trimmed using Audacity (ver. 2.3) so that each sound clip’s duration was 300 ms. To increase the variety of the acoustic properties while preserving the phonetic features, the Voicemod program was used to transform the female voice into a voice with higher or lower frequencies, resulting in 12 sounds. The volumes of these 12 sounds were equalized to have the same root mean square contrast. The temporal contours of the fundamental frequencies (F0s) of the 12 sounds (/i1/, /u1/, /i4/, and /u2/) as a function of time (ms) are plotted in Figure 2A. Figure 2B shows the mean and standard deviation across the time of the F0, the first formant (F1), the second formant (F2), and the third formant (F3) of the 12 sounds.

Three pairs of angular and rounded visual patterns were adopted from previous studies (Köhler, 1929; Ramachandran and Hubbard, 2001; Bremner et al., 2013). The size of each pattern was 9.3° × 9.3° in visual angle, and each pattern was presented in one of four orientations. The two response keys were labeled using color stickers to ensure that the letters’ shapes would not influence the participants’ performance.

### 2.3. Explicit matching tasks

All of the participants conducted the explicit matching tasks either before or after the main experiment. There were two types of matching tasks. In the sound matching task, in each trial, a tonal vowel and a pair of angular and rounded patterns were presented on the monitor side by side, randomly on the left and right. The participants had to determine which of the two patterns better matched the tonal vowel. The visual patterns disappeared after the participants responded, and the next trial started after 1,000 ms. There were 36 trials (4 tonal vowels * 3 voices * 3 pairs of visual patterns) presented in a randomized order.

In the shape matching task, in each trial, we presented a visual pattern in the center of the monitor and then a pair of sounds (/i1/ and /u1/, or /i4/ and /u2/ in a low, medium, or high frequency) in a random order. The participants had to determine which of the two sounds better matched the visual pattern. The visual pattern disappeared after the participants responded, and the next trial started after 1,000 ms. There were 36 trials (6 visual shapes * 2 pairs of tonal vowels * 3 voices), and their presentation order was randomized. We presented the two matching tasks in a randomized order across participants. The procedure took approximately 10 min to complete.

## 3. Experiment 1: implicit association test

In Experiment 1, the participants performed the IAT. In every trial, they had to discriminate the sounds as either vowel /i/ or /u/ or the visual patterns as either angular or rounded rapidly and accurately by pressing predesignated keys.

### 3.1. Design and procedure

The IAT consisted of two sessions, one to test the sound pair /i1/ and /u1/ and the other to test the sound pair /i4/ and /u2/. We conducted these two sessions in an evenly distributed order between the participants. Each session consisted of five blocks: three for training (blocks 1, 2, and 4) and two for the main experiment (blocks 3 and 5). In block 1, the participants practiced to rapidly categorize visual patterns by pressing predesignated keys (e.g., angular shape: left key, rounded shape: right key). In block 2, they practiced to rapidly discriminate the auditory stimuli by pressing the same predesignated keys (e.g., /i1/: left key, /u1/: right key). In block 3, we measured the RTs of congruent audiovisual mappings (e.g., angular shape and /i1/: left key, rounded shape and /u1/: right key). In block 4, participants practiced with the visual patterns again, but we swapped the stimulus–response mappings from block 1 (e.g., rounded shape: left key, angular shape: right key). Finally, we used block 5 to measure the RTs of incongruent audiovisual mappings (e.g., rounded shape and /i1/: left key, angular shape and /u1/: right key). Throughout the experiment, the vowel /i/ always corresponded to the left key and the vowel /u/ always corresponded to the right key regardless of lexical tone. We balanced the presentation order of blocks 1 and 3 and blocks 4 and 5 between participants to control for the potential effect of practicing the IAT.

In the visual and auditory training blocks, we presented six patterns or six sounds (2 tonal vowels * 3 voices) 6 times, resulting in 36 trials. In the congruent and incongruent blocks, we presented each pattern and sound 12 times, resulting in 144 trials. To ensure that the terminology did not prime participants regarding crossmodal association, we showed them two categories of patterns instead of using verbal description when delivering the experimental instruction. At the beginning of each block, the response key (left or right) for each category of patterns and/or sounds was assigned.

In each trial, one stimulus–either a pattern or sound–was presented on the screen or through the headphones. Each stimulus was presented for 300 ms. When the auditory stimulus was presented, a fixation cross was presented on the screen. The participants had to categorize the presented stimulus as quickly and correctly as possible by pressing the corresponding key. The participants were instructed to press the left and right keys (A and L keys on the keyboard) using their left and right index finger, respectively. In the training blocks, an X was presented in the center of the screen when the response was incorrect; however, there was no feedback in the congruent and incongruent blocks. There was no time limit for participants to respond. The IAT task took around 60 min to complete.

### 3.2. Data analysis

We analyzed the data in the congruent and incongruent blocks. For each participant, we defined the trials with RT less than 100 ms or greater than mean plus three standard deviations (SDs) as outliers and excluded them from further analyses. In each experiment, however, there was no trial with RT shorter than 100 ms, so the excluded trials were all attributed to long responses (i.e., longer than mean + 3SDs). We report the accuracy, RT, and their associated analyses including lexical tones as a within-participant factor in Supplementary materials. In the main text, we combined the two sessions of vowels with different lexical tones.

To quantify the magnitude of the congruency effects in terms of cross-modal correspondences, we computed the *d-score* by taking the RT difference (subtract the mean RT in the congruent condition from the mean RT in the incongruent condition) and then dividing it by the pooled standard deviation. Therefore, the larger the RT difference or the smaller the RT variability, the larger the d-score. Given the prior assumption that the RT should be longer in the incongruent than in the congruent condition, we conducted a one-sample *t*-test (one-tailed) to determine whether the d-score was significantly greater than zero, indicating a significant congruency effect. We computed the d-score for each stimulus modality (visual or auditory), which we would then use to compare the congruency effect across experiments. We compared the visual and auditory d-scores using a paired *t*-test to investigate the symmetry of sound–shape correspondences.

To investigate the stage of information processing at which sound–shape correspondences occurred, we ran a bin analysis of RTs (see De Jong et al., 1994; Vallesi et al., 2005; Parise and Spence, 2012) by separating the RT data in each modality and congruency condition into five time bins, from fastest to slowest, for each participant. We then used the RTs in each time bin to compute the d-score in each time bin. We initially compared these d-scores to zero using one-sample *t*-tests; however, because the d-scores in the five time bins were related rather than independent, we set the criterion of statistical significance at *p* < 0.01 to avoid inflating α. To compare the congruency effect between modalities across time bins, we submitted the d-scores to a two-way repeated-measure ANOVA on the within-participant factors of modality (visual and audition) and bin (1 to 5). We used a Greenhouse–Geisser correction when the sphericity assumption was violated. *Post hoc* comparisons for time bins were pairwise *t*-tests with Bonferroni corrections. The data in Experiments 2 and 3 underwent the same analysis procedure, but we obtained only auditory d-scores and visual d-scores, respectively.

### 3.3. Results

**Figure 3:**
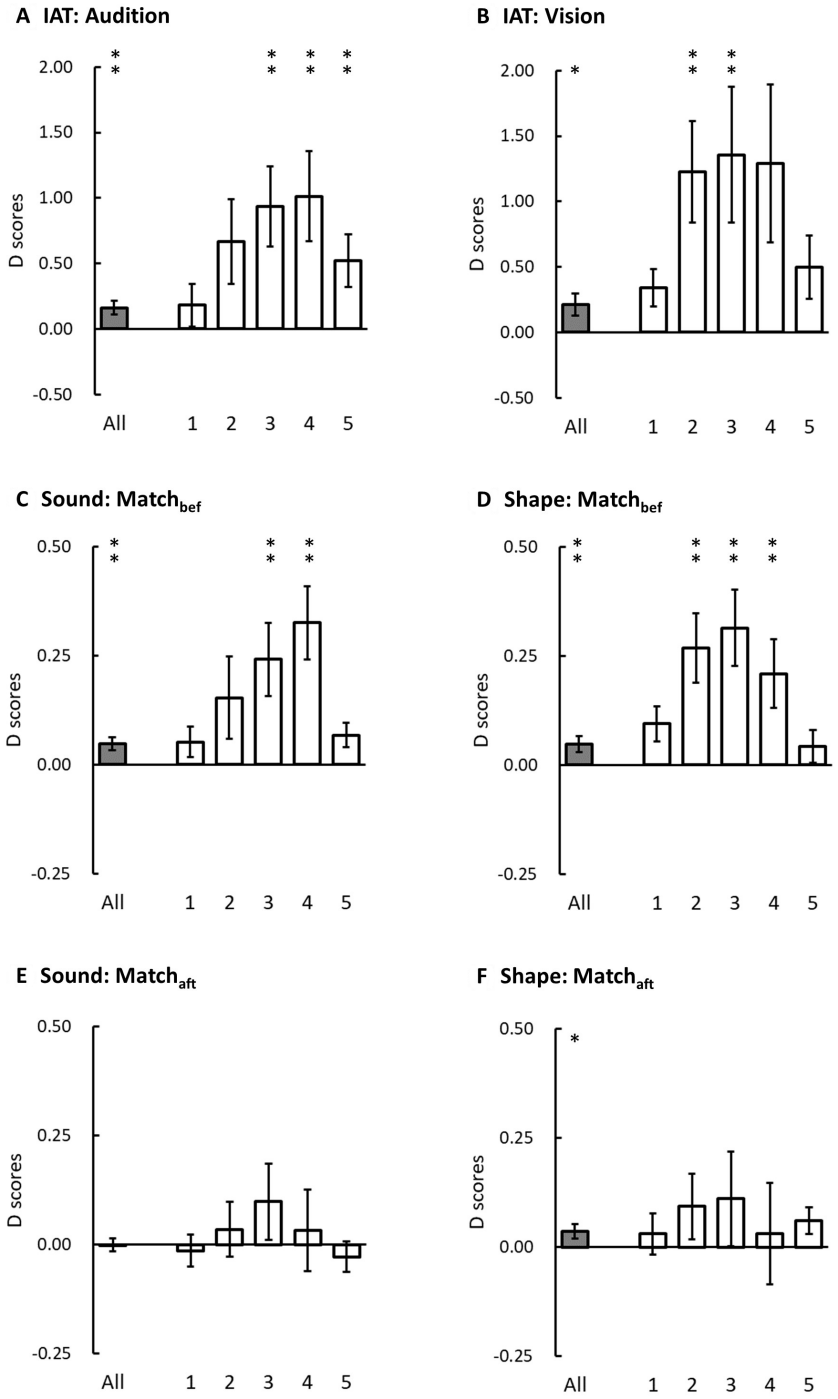
Overall d-scores (grey bars) and the d-scores in each time bin (white bars) in three experiments. The left panels are auditory d-scores and the right panels are visual d-scores. **(A,B)** present auditory and visual d-scores in the IAT in Experiment 1. **(C,E)** present auditory d-scores in the sound classification task when the participants performed the explicit matching tasks either before (Match_bef_) or after (Match_aft_) the main task in Experiments 2A and 2B. **(D,F)** present the visual d-scores in the shape classification task when the participants performed the explicit matching tasks before (Match_bef_) or after (Match_aft_) the main task in Experiments 3A and 3B. The error bars represent one standard error of the mean. The asterisks indicate statistical difference from zero (* denotes *p* < 0.05 and ** denotes *p* < 0.01 before Bonferroni corrections; note that the significant criterion for the d-scores in each time bin was set at *p* < 0.01).

The overall accuracy was 96.61 ± 0.43% (mean ± SE). We removed the 3.39% incorrect trials and 2.30% trials as RT outliers from the computations of d-scores. The auditory and visual d-scores in the IAT demonstrated reliable congruency effects (Figures 3A,B, respectively): auditory d-score = 0.16 (*SE* = 0.05, *t*(23) = 2.99, *p* = 0.003, *Cohen’s d* = 0.61) and visual d-score = 0.21 (*SE* = 0.08, *t*(23) = 2.52, *p* = 0.01, *Cohen’s d* = 0.51). Furthermore, the visual and auditory d-scores were similar (*t*(23) = 0.80, *p* = 0.43, *Cohen’s d* = 0.16), suggesting a similar magnitude of congruency effect between vision and audition.

When we computed the d-scores individually in each time bin, the results demonstrated that the auditory d-scores were greater than zero in the third, fourth, and fifth bins (all *t*(23) > 2.59, *p*s ≤ 0.008, *Cohen’s d* > 0.52) but not in the first two bins (both *t*(23) < 2.05, *p*s ≥ 0.025, *Cohen’s d* < 0.42); the visual d-scores were greater than zero in the second and third bins (both *t*(23) > 2.61, *p*s ≤ 0.008, *Cohen’s d* > 0.53) but not in the first, fourth, and fifth bins (all *t*(23) < 2.34, *p*s ≥ 0.014, *Cohen’s d* < 0.48). We submitted these d-scores to a two-way ANOVA on the factors of modality and bin. The main effect of bin was significant (*F*(2.07, 47.63) = 5.06, *p* = 0.01, *η_p_^2^* = 0.18). The *post hoc* tests showed that the d-score was significantly lower in the first bin than in the second and third bins (both *t*(23) > 3.12, *p*s ≤ 0.048, *Cohen’s d* > 0.63). However, the main effect of modality (*F*(1, 23) = 1.08, *p* = 0.31, *η_p_^2^* = 0.05) and the interaction between modality and bin (*F*(1.79, 41.07) = 1.05, *p* = 0.38, *η_p_^2^* = 0.04) were not significant. Taken together, the d-score was not significant in the first bin, and it was significantly smaller than in the central bins, and therefore did not support an early onset of the congruency effect. The effect of modality did not reach a significant level, suggesting a symmetrical modulation of crossmodal correspondences (Evans and Treisman, 2010; Parise and Spence, 2012).

## 4. Experiments 2A and 2B: sound classification task

In Experiments 2A and 2B, the participants had to discriminate the sounds as either the vowel /i/ or /u/ rapidly and accurately irrespective of the lexical tones; meanwhile, a task-irrelevant visual pattern was presented as a distractor. In Experiment 2A, the participants performed the explicit matching tasks *before* the sound classification task (called the *Match_bef_* condition hereafter); in Experiment 2B, the participants performed the explicit matching tasks *after* the sound classification task (called *Match_aft_* condition hereafter).

### 4.1. Design and procedure

The sound classification task comprised two sessions, one to test the /i1/ and /u1/ pair and the other to test the /i4/ and /u2/ pair. We evenly distributed the order of these two sessions between participants.

In each trial, the sound was presented for 300 ms; meanwhile, either a congruent visual shape (e.g., an angular shape for /i1/) or an incongruent visual shape (e.g., a rounded shape for /i1/) was presented. Participants had to categorize the sound as /i/ or /u/ (irrespective of the lexical tones) as rapidly and correctly as possible by pressing predesignated keys (A and L keys on the keyboard, counterbalanced between participants) using the left and right index finger, respectively. There was no time limit for participants to respond. After the response, an inter-trial interval (ITI) ranging from 500 ± 50 ms was presented and followed by the next trial. No clue was provided at the beginning of each trial, so the participants had to concentrate on the task continuously. Each session consisted of 400 trials, and the participants had a short break after every 100 trials. The participants practiced a few trials and ensured that they understood the task before the main experiment. The experiment took around 40 min to complete.

### 4.2. Results

In Experiment 2A (the Match_bef_ condition, Figure 3C), the overall accuracy was 97.68 ± 0.34%. There were 2.32% incorrect trials and 2.13% of trials as RT outliers removed from the computation of d-scores. The auditory d-score was 0.05 (*SE* = 0.02), which was significantly greater than zero (*t*(23) = 3.12, *p* = 0.002, *Cohen’s d* = 0.64), demonstrating a significant congruency effect induced by visual distractors. When we separately computed the d-scores in the five time bins, the d-score was significantly greater than zero in the third and fourth bins (both *t*(23) > 2.88, *p*s ≤ 0.004, *Cohen’s d* > 0.58) but not in the first, second, or fifth bins (all *t*(23) ≤ 2.45, *p*s ≥ 0.011, *Cohen’s d* < 0.50). We submitted the auditory d-scores in the five time bins to a one-way ANOVA on the factor of bin. The main effect was significant (*F*(2.29, 52.68) = 5.80, *p* = 0.004, *η_p_^2^* = 0.20). The *post hoc* tests showed that the d-score was significantly smaller in the first and fifth bins than in the fourth bin (both *t*(23) > 3.44, *p* ≤ 0.022, *Cohen’s d* > 0.70).

In Experiment 2B (the Match_aft_ condition, Figure 3E), the overall accuracy was 97.43 ± 0.35%. There were 2.57% incorrect trials and 1.89% of trials as RT outliers removed from the computation of d-scores. The overall d-score was −0.0003 (*SE* = 0.01), which was not significantly different from zero (*t*(23) = −0.02, *p* = 0.49, *Cohen’s d* = 0.004). Consistently, none of the d-scores in the five time bins were significantly different from zero (all *t*(23) ≤ 1.14, *p*s > 0.13, *Cohen’s d* < 0.23).

To investigate the influences of the order of the explicit matching tasks either before or after the sound classification task, we compared the d-scores in Experiments 2A and 2B. The auditory d-score was significantly larger in the Match*_bef_* than in the Match*_aft_* condition (two-tailed, *t*(46) = 2.25, *p* = 0.03, *Cohen’s d* = 0.65), suggesting that performing the explicit matching tasks before sound classification task rather than the reversed order induced a larger congruency effect.

## 5. Experiments 3A and 3B: shape classification task

In Experiments 3A and 3B, the participants had to discriminate a visual pattern rapidly and accurately as belonging to the angular or rounded category; meanwhile, a task-irrelevant sound was presented as a distractor. In Experiment 3A, the participants performed the explicit matching tasks just before the shape classification task (i.e., the Match_bef_ condition); in Experiment 3B, the participants performed the explicit matching tasks after the shape classification task (i.e., the Match_aft_ condition).

### 5.1. Design and procedure

The shape classification task comprised two sessions. In one session, either the tonal vowel /i1/ or /u1/ was presented as distractor; in the other session, either the tonal vowel /i4/ or /u2/ was presented as distractor. The order of these two sessions was evenly distributed between participants.

In each trial, the shape, either an angular or rounded pattern, was presented in the center of the monitor for 300 ms; meanwhile, either a congruent or incongruent sound (see above) was presented. The participants were required to categorize the shape as an angular or rounded pattern as rapidly and correctly as possible by pressing predesignated keys (A and L keys on the keyboard, evenly distributed between participants) using the left and right index finger, respectively. Other details were the same as in the sound classification task.

### 5.2. Results

In Experiment 3A (the Match_bef_ condition, Figure 3D), the overall accuracy was 96.10 ± 0.58%. There were 3.90% incorrect trials and 1.94% of trials as RT outliers removed from the computation of d-scores. The visual d-score was 0.05 (*SE* = 0.02), which was significantly greater than zero (*t*(23) = 2.60, *p* = 0.008, *Cohen’s d* = 0.53). Follow-up bin analysis demonstrated that the visual d-scores in the second, third, and fourth bins were significantly greater than zero (all *t*(23) > 2.64, *p*s < 0.007, *Cohen’s d* > 0.53) but not in the first and fifth bins (both *t*(23) ≤ 2.33, *p*s > 0.014, *Cohen’s d* < 0.48). The visual d-scores in the five time bins were submitted to a one-way ANOVA on the factor of bin. The main effect was significant (*F*(2.58, 59.41) = 4.64, *p* = 0.008, *η_p_^2^* = 0.17). The *post hoc* tests showed that the d-score was significantly smaller in the first than in the second bin (*t*(23) = 3.18, *p* = 0.04, *Cohen’s d* = 0.64).

In Experiment 3B (the Match_aft_ condition, Figure 3F), the overall accuracy was 95.63 ± 0.56%. There were 4.37% incorrect trials and 1.72% of trials as RT outliers removed from the computation of d-scores. The visual d-score was 0.04 (*SE* = 0.02), which was significantly greater than zero (*t*(23) = 2.15, *p* = 0.02, *Cohen’s d* = 0.44). However, the visual d-scores in the five time bins did not show a significant difference from zero (all *t*(23) ≤ 2.00, *p*s > 0.029, *Cohen’s d* < 0.41). The visual d-scores did not show a significant difference between the Match*_bef_* and Match*_aft_* conditions (two-tailed, *t*(46) = 0.52, *p* = 0.61, *Cohen’s d* = 0.15).

## 6. Comparisons of experiments 1, 2, and 3

### 6.1. The magnitude of auditory and visual congruency effect

To compare the magnitude of congruency effect in the sound and shape classification tasks, we submitted the d-scores in Experiments 2 and 3 to a two-way ANOVA on the between-participant factors of modality (auditory or visual) and order (Match_bef_ or Match_aft_). The results showed that the d-score was similar in the Match_bef_ and Match_aft_ conditions (0.05 vs. 0.02, *F*(1, 92) = 3.46, *p* = 0.066, *η_p_^2^* = 0.04). The main effect of modality was not significant (auditory: 0.02, visual: 0.04, *F*(1, 92) = 1.24, *p* = 0.27, *η_p_^2^* = 0.01), nor was the interaction between modality and order (*F*(1, 92) = 1.15, *p* = 0.29, *η_p_^2^* = 0.01). Therefore, the magnitude of congruency effect was similar irrespective of the order of explicit and implicit tasks, and it was similar in the two modalities, consistent with the results in IAT (see Results in Experiment 1).

### 6.2. Modulation of the congruency effect by task relevance

In Experiment 1, the visual and auditory modalities were task-relevant in the IAT; in Experiments 2 and 3, only the auditory or visual modality was task-relevant in the sound and shape classification tasks, respectively. By comparing the d-scores obtained from the IAT to those of the speeded classification tasks, we could determine whether the magnitude of the congruency effect depended on the task relevance of sensory modalities. To be fair, we only compared the experiments in which the explicit matching tasks were performed *after* the main experiment (i.e., Experiments 1, 2B, and 3B). The auditory d-score was higher in the IAT than in the sound classification task (two-tailed, *t*(46) = 2.89, *p* = 0.006, *Cohen’s d* = 0.83). Similarly, the visual d-score was higher in the IAT than in the shape classification task (two-tailed, *t*(46) = 2.06, *p* = 0.045, *Cohen’s d* = 0.59). Therefore, the congruency effect was more pronounced when both modalities rather than only one were task-relevant.

### 6.3. The results of the explicit matching tasks

**Figure 4:**
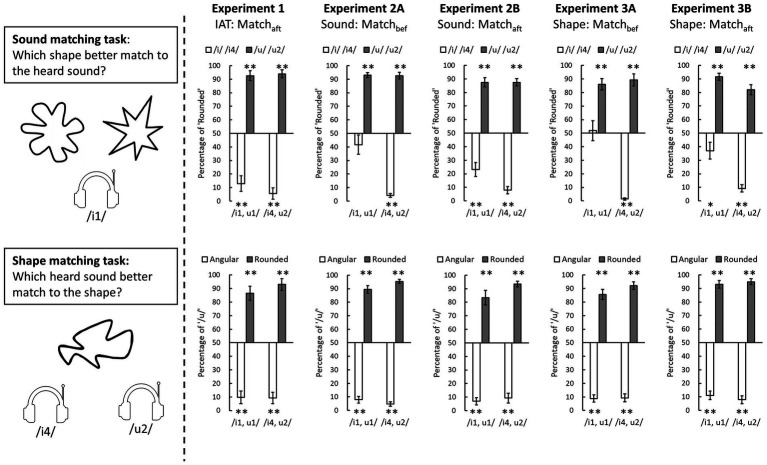
The results of explicit matching tasks in three experiments. The sound matching performance is presented in the upper panels and the shape matching performance in the lower panels. The white bars represent the tonal vowels /i1/ or /i4/ in the sound matching task or angular patterns in the shape matching task; the grey bars represent the tonal vowel /u1/ or /u2/ in the sound matching task or rounded patterns in the shape matching task. The error bars represent one standard error of the mean (* denotes *p* < 0.05, ** denotes *p* < 0.01 of the result of a one-sample *t*-test compared to 50%).

The matching tendency in the explicit matching tasks, as represented by the proportions that participants matched each tonal vowel to rounded patterns or the proportion that they matched each visual pattern to the vowel /u/, are reported in Figure 4. To examine any consensual matching in each condition, the proportion of matching was compared to 50% using a one-sample *t*-test (two-tailed).

In the sound matching task (upper panels in Figure 4), the participants tended to match the vowel /i/ to angular patterns and the vowel /u/ sound to rounded patterns for most of the tonal vowels (*t*(23) ≥ 2.07, *p*s ≤ 0.05, *Cohen’s d* > 0.42) except the /i1/ in Experiments 2A (*t*(23) = 1.67, *p* = 0.26, *Cohen’s d* = 0.34) and 3A (*t*(23) = 0.25, *p* = 0.80, *Cohen’s d* = 0.05); here both were the condition where the explicit matching tasks had been performed before the main experiment. Even though the participants’ matching between /i1/ and angular pattern was ambiguous, the contrast to the matching between /u1/ and rounded pattern remained significant (Experiment 2A: *t*(23) = 7.33, *p* < 0.001, *Cohen’s d* = 1.50; Experiment 3A: *t*(23) = 4.58, *p* < 0.001, *Cohen’s d* = 0.93). The result that the vowel /i1/ was reliably matched to angular patterns only in the Match_aft_ condition (Experiments 1, 2B, and 3B) may suggest that the repeated presentation of the tonal vowels and visual patterns in the preceding main task would improve the reliability of the sound-shape correspondences when the participants responded in the explicit matching tasks.

In the shape matching task (lower panels in Figure 4), the results showed that the participants reliably match rounded patterns to /u/ while matching angular patterns to /i/ (all *t*(23) ≥ 6.13, *p*s ≤ 0.001, *Cohen’s d* > 1.25). Compared to the sound matching task, the judgments in the shape matching seemed more reliable and insensitive to the prior exposure of these stimuli.

## 7. Discussion

In the present study, we examined the automaticity and symmetry of a classic example of crossmodal correspondences, that is, the sound–shape correspondences between vowels /i/ and /u/, and angular and rounded patterns, respectively. We utilized two implicit tasks, the IAT and speeded classification tasks, and the commonly used explicit matching tasks to explore this issue. In the IAT, in which the participants had to categorize a sound or pattern rapidly and accurately, the significant congruency effect was demonstrated in the auditory and visual modalities. In the sound classification task, we only observed the significant congruency effect induced by visual distractors when the explicit matching tasks were performed before rather than after the sound classification task; in contrast, in the shape classification task, we observed the significant congruency effect induced by auditory distractors when the explicit matching tasks were performed either before or after the shape classification task. Further bin analysis of RTs demonstrated that we did *not* observe the congruency effect in the first bin but observed it in the later bins. Finally, when we compared the IAT and speeded classification tasks, the congruency effect was more pronounced in the IAT, suggesting that the task relevance of modalities is critical for the encoding of sound–shape correspondences. Combined, these results of congruency effects suggest that the sound–shape correspondences tested here did not occur automatically.

The congruency effect of sound–shape correspondences did not demonstrate unequivocal evidence of automaticity. The auditory and visual congruency effects were significant in most experiments (except in Experiment 2B), but the congruency effect was never significant in the first time bin. This result contrasts with the observation of Parise and Spence (2012), who demonstrated the congruency effect in the first time bin and suggested that sound–shape associations were encoded automatically according to the speed criterion. In addition, the congruency effect was more pronounced when the auditory and visual modalities were task-relevant in the IAT than when only one modality was task-relevant in the speeded classification tasks. This result suggests that encoding the sound–shape correspondences during information processing depended on whether visual and auditory modalities were related to the goal at hand to a certain extent, thereby violating the involuntariness (unintentionality) criterion. These two pieces of evidence therefore do not support the automatic processing of sound–shape correspondences. Note that the auditory congruency effect was only significant when the explicit matching tasks were performed before (Experiment 2A) rather than after (Experiment 2B) the sound classification task; however, we did not observe this order difference in the visual congruency effect (Experiments 3A and 3B). Critically, a further comparison by combining Experiments 2 and 3 did not demonstrate a reliable order difference, either. Therefore, despite the participants’ explicit matching of the sound-shape correspondences may facilitate the informativeness of the distractor to target in terms of their congruency, it does not appear to be decisive in the automaticity of the sound-shape correspondences.

Evidence of the symmetrical modulations of sound–shape correspondences in either modality was apparent. When we compared the magnitudes of the auditory and visual congruency effects, we found no significant difference in the IAT or speeded classification tasks. In addition, the results of bin analysis of RTs in the IAT demonstrated a similar pattern of visual and auditory congruency effect across time bins. Taken together, the modulations of sound–shape correspondences were symmetrical in terms of the magnitude and onset of the congruency effects, consistent with Evans and Treisman (2010) and Parise and Spence (2012).

We quantified the congruency effect of sound–shape correspondences by calculating d-scores in the IAT and speeded classification tasks. Even though the magnitude of the congruency effect is presumed to reflect the strength of associations between the sounds and shapes tested in the current study, the underlying mechanism in the two paradigms is substantially different. In the IAT, the participants responded to stimuli in the auditory and visual modalities and tried to associate the sound and shape that were mapped to the same response to speed up the responses and reduce errors. This task-demanded association should be easier to establish in the congruent block, in which the corresponding sounds and shapes were mapped to the same response, than in the incongruent block, in which the contrasting sounds and shapes were mapped to the same response. Therefore, factors that help establish new associations, such as learning and practicing, may influence the magnitude of the congruency effect in the IAT (Greenwald et al., 1998; Hussey and De Houwer, 2018). In the speeded classification task, in contrast, the participants had to respond selectively to the modality in which the target was presented while ignoring (or inhibiting) the other modality in which the distractor was presented; that is, the participants tried to dissociate the simultaneously presented sound and shape. If they failed to inhibit the distractor but processed it to a certain level, it may have facilitated (or interfered with) the processing of the target when they correspond (or contrast). Therefore, the participant’s execution of selective attention or the stimulus’s complexity (determining the perceptual load of processing) may influence the congruency effect in the speeded classification tasks (Parise and Spence, 2012; Molloy et al., 2015). Given that the task relevance of sensory modalities and intention to relate the stimuli (associate vs. dissociate) differed in the IAT and speeded classification tasks, utilizing either experimental paradigm alone is perhaps insufficient to examine the automaticity of the crossmodal correspondences.

It should be noted that we do not intend to generalize our conclusion to all types of crossmodal correspondences. In fact, based on our previous studies, we postulate that the sound–shape correspondences occur at the “mid-level” processing, a higher level than the crossmodal correspondences of simple features (such as the pitch–size correspondences in Figure 1A). For example, in the visual modality, the process of perceptual grouping from elements to a global contour is essential in the sound–shape correspondences (Chen et al., 2021). In addition, not only the acoustic features (Knoeferle et al., 2017) but also the phonemes that are perceptually invariant (Shen et al., 2022) seem to drive the sound–shape correspondences. It has been suggested that crossmodal correspondences can occur at multiple levels of information processing (Spence, 2011), and it is plausible that higher-level correspondences may be more susceptible to cognitive factors and therefore more controllable than lower-level correspondences.

In conclusion, we utilized two implicit tasks—IAT and speeded classification tasks—to examine the automaticity and symmetry of sound-shape correspondences in modulating people’s speeded behavioral performance. Given that the congruency effects occurred with later responses and they were susceptible to the task relevance of sensory modalities, we suggest that the associations between sounds and shapes were not encoded automatically. In addition, the magnitude and onset of the visual and auditory congruency effects were comparable, suggesting a symmetrical modulation of sound-shape correspondence between sensory modalities once it occurred. The current study demonstrates a methodological solution when one aims to examine the automaticity and symmetry of cross-modal correspondences carefully (see Spence and Deroy, 2013, for a review and discussion).

## Data availability statement

The raw data supporting the conclusions of this article will be made available by the authors, without undue reservation.

## Ethics statement

The studies involving human participants were reviewed and approved by National Cheng Kung University research ethics committee for human behavioral sciences (NCKU HREC-E-110-460-2). The patients/participants provided their written informed consent to participate in this study.

## Author contributions

P-CH and Y-CC: conception and design of study, interpretation of data, and writing the article. P-CH: acquisition and analysis of data. All authors contributed to the article and approved the submitted version.

## Funding

This work was supported by the Ministry of Science and Technology in Taiwan (MOST 108-2410-H-006-041-MY2 to P-CH and MOST 110-2423-H-715-001-MY3 to Y-CC), and MacKay Medical College (MMC-RD-110-1B-P024 to Y-CC).

## Conflict of interest

The authors declare that the research was conducted in the absence of any commercial or financial relationships that could be construed as a potential conflict of interest.

## Publisher’s note

All claims expressed in this article are solely those of the authors and do not necessarily represent those of their affiliated organizations, or those of the publisher, the editors and the reviewers. Any product that may be evaluated in this article, or claim that may be made by its manufacturer, is not guaranteed or endorsed by the publisher.
