# Supplementary material for: Examining the automaticity and symmetry of sound–shape correspondences
Source: Front Psychol. 2023 Jun 5;14:1172946. doi: 10.3389/fpsyg.2023.1172946 (PMC10277733; doi:10.3389/fpsyg.2023.1172946)
Supplement: Supplementary file 1 [file Data_Sheet_1.pdf]

Supplementary Material

Examining the automaticity and symmetry of sound–shape correspondences

Yi-Chuan Chen, Pi-Chun Huang *

*** Correspondence:** Pi-Chun Huang: pichun_huang@mail.ncku.edu.tw

1. Experiment 1: Implicit Association Task (IAT)

The mean accuracy (the left panels in Figures S1A, S1B) was submitted to a three-way repeated measure analysis of variance (ANOVA) on the within-subject factors of congruency (congruent vs. incongruent), lexical tone (/i1, u1/ vs. /i4, u2/) and stimulus type (sound or shape). The main effects of congruency and modality were significant: The accuracy was higher in the congruent than in the incongruent condition (97.32% vs. 95.91%, $F(1,23) = 8.86$, $p = 0.007$, $\eta_p^2 = 0.28$), and also higher when the target was a sound rather than a shape (97.60% vs. 95.63%, $F(1,23) = 17.33$, $p <0 .001$, $\eta_p^2 = 0.43$). The main effect of lexical tone was not significant ($F(1,23) = 0.53$, $p = 0.47$, $\eta_p^2 = 0.02$). None of the interaction was significant (all $F(1,23) < 3.27$, *p*s > 0.083, $\eta_p^2 < 0.13$).

The mean response time (RT, the right panels in Figures S1A, S1B) was submitted to a three-way repeated measure ANOVA on the same factors. The results demonstrated significant main effects of congruency and modality: The RT was shorter in the congruent than in the incongruent trials (670 ms vs. 709 ms, $F(1,23) = 7.33$, $p = 0.013$, $\eta_p^2 = 0.24$), and the RT was shorter when the target was a shape rather than a sound (621 ms vs. 759 ms, $F(1,23) = 108.29$, $p < 0.001$, $\eta_p^2 = 0.83$). The main effect of lexical tone was not significant ($F(1,23) = 0.09$, $p = 0.77$, $\eta_p^2 = 0.004$), nor was any level of interaction (all $F(1,23) < 0.83$, *p*s > 0.37, $\eta_p^2 < 0.04$). Taken together, the accuracy was higher and RT was shorter

in the congruent condition than in the incongruent condition, suggesting that there was no speed-accuracy trade-off regarding the factor of congruency.

The *d-score* in each condition (Figures S2A, S2B) was submitted to a one-sample *t*-test. The d-scores in all four conditions were significantly greater than zero (all $t(23) > 1.92$, *p*s < 0.034, *Cohen's d* > 0.39). The d-scores were then submitted to a two-way repeated-measure ANOVA with the within-subject factors of lexical tone and stimulus type. The results demonstrated that none of the main effects and interaction was significant (all $F(1,23) < 0.06$, *ps* > 0.80, $\eta_p^2 < 0.03$).

2. Experiment 2: Sound classification task

The mean accuracy (the left panels in Figures S1C, S1E) was submitted to a three-way ANOVA with two within-subject factors of congruency (congruent vs. incongruent), lexical tone (/i1, u1/ vs. /i4, u2/), and a between-participant factor of the order of matching task. The main effect of congruency was significant that the accuracy was higher in the congruent than in the incongruent trials (97.72% vs. 97.38%, $F(1,46) = 5.41$, $p = 0.024$, $\eta_p^2 = 0.11$). Neither other main effects nor any interactions reached statistical significance (all $F(1,46) < 3.11$, *p*s > 0.085, $\eta_p^2 < 0.06$).

The mean RT (the right panels in Figures S1C, S1E) was submitted to a three-way ANOVA on the same factors. The main effect of lexical tone was significant that the RT was shorter in /i1, u1/ than in the /i4, u2/ trials (527 ms vs. 545 ms, $F(1,46) = 4.18$, $p = 0.046$, $\eta_p^2 = 0.08$). No other main effect or interaction was significant (all $F(1,46) < 3.15$, *ps* > 0.083, $\eta_p^2 < 0.06$).

The d-score in each condition (Figures S2C, S2E) was submitted to a one-sample *t*-test. The d-score was significantly greater than zero only in the $Match_{bef}$ condition for /i1, u1/ ($t(23) = 3.54$, $p < 0.001$, *Cohen's d* = 0.72), but not in the other three conditions (all $t(23) < 1.20$, *p*s > 0.12, *Cohen's d* < 0.24). When the d-scores were submitted to a two-way ANOVA on the within-participant factor of lexical tone and the between-participant factor of the order of matching task, none of main effects and interaction was significant (all $F(1,46) < 2.90$, *ps* > 0.095, $\eta_p^2 < 0.06$). Taken together, the effect of lexical tone was not reliable in the implicit tasks (similar results in the IAT, Shang and Style, 2023).

3. Experiment 3: Shape classification task

The mean accuracy (the left panels in Figures S1D, S1F) was submitted to a three-way ANOVA with two within-subject factors of congruency (congruent vs. incongruent), lexical tone (/i1, u1/ vs. /i4, u2/), and a between-participant factor of the order of matching task. No main effect or any interaction was significant (all $F(1,46) < 3.62$, ps > 0.063, $\eta_p^2 < .073$). The mean RT (right panels in Figures S1D, S1F) was submitted to a three-way ANOVA on the same factors and also showed no significance for every main effect and interaction (all $F(1,46) < 4.02$, ps ≥ 0.0507, $\eta_p^2 < 0.08$).

The d-score in each condition (Figures S2D, S2F) was submitted to a one-sample *t*-test. The d-scores in both lexical tone conditions were greater than zero in the $Match_{bef}$ condition (/i1, u1/: $t(23) = 2.30$, $p = 0.015$, *Cohen's d* = 0.46; /i4, u2/: $t(23) = 1.89$, $p = 0.036$, *Cohen's d* = 0.39). However, the d-scores were not significant compared to zero in the $Match_{aft}$ conditions (both $t(23) < 1.12$, ps > 0.13, *Cohen's d* < 0.23). When the d-scores were submitted to a two-way ANOVA on the within-participant factor of lexical tone and the between-participant factor of the order of matching task, none of the main effect and interaction was significant (all $F(1,46) < 1.32$, ps > 0.255, $\eta_p^2 < 0.028$). Hence, the effect of lexical tone was not reliable in the implicit tasks (similar results in the IAT, Shang and Style, 2023).

4. Reference

Shang, N. & Styles, S. J. (2023). Implicit Association Test (IAT) Studies Investigating Pitch-Shape Audiovisual Cross-modal Associations Across Language Groups. *Cogn Sci*, 47(1), e13221. doi: 10.1111/cogs.13221

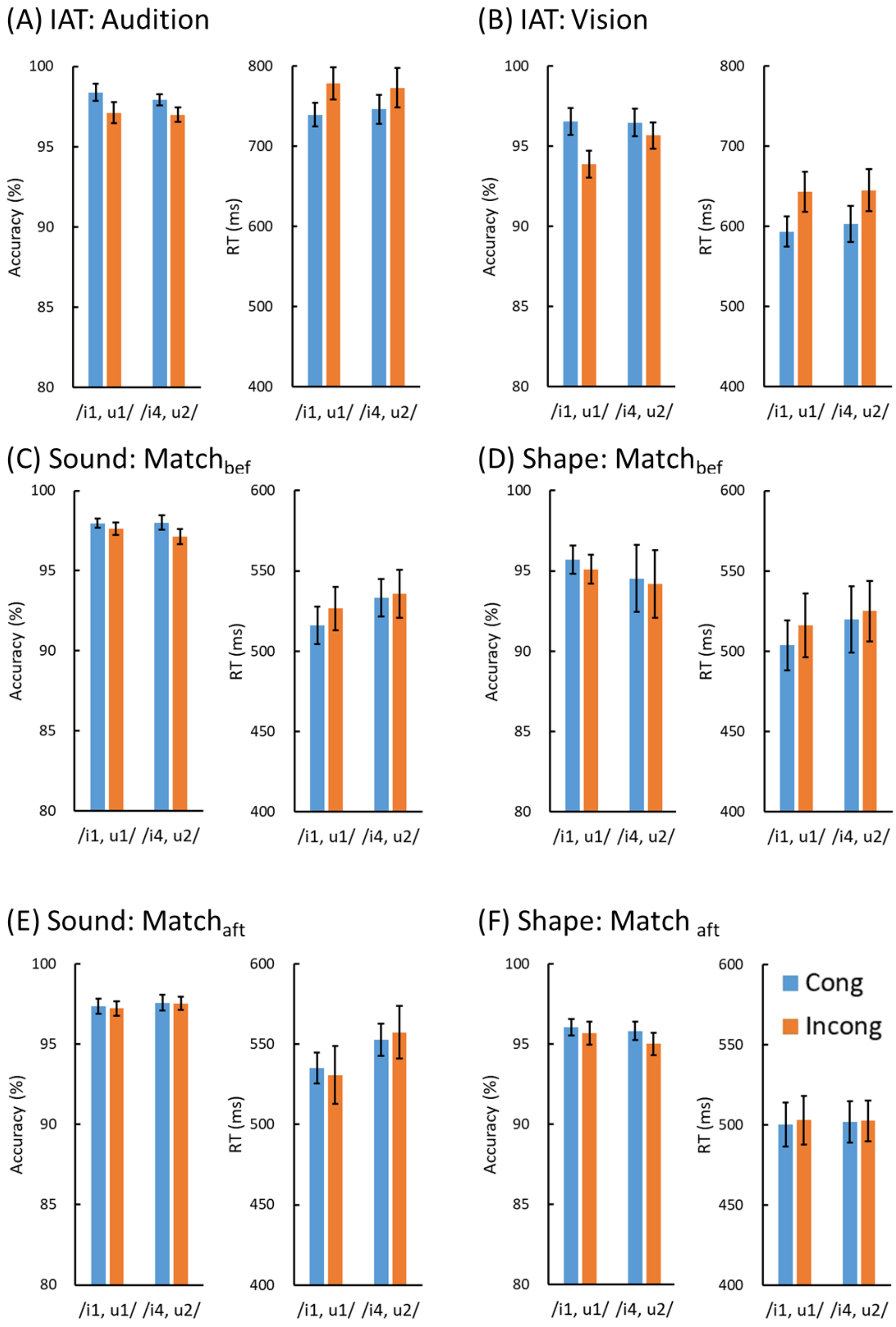

Figure S1. Mean accuracy and RT in Experiments 1 to 3. The error bars represent ±1 standard error of the mean.

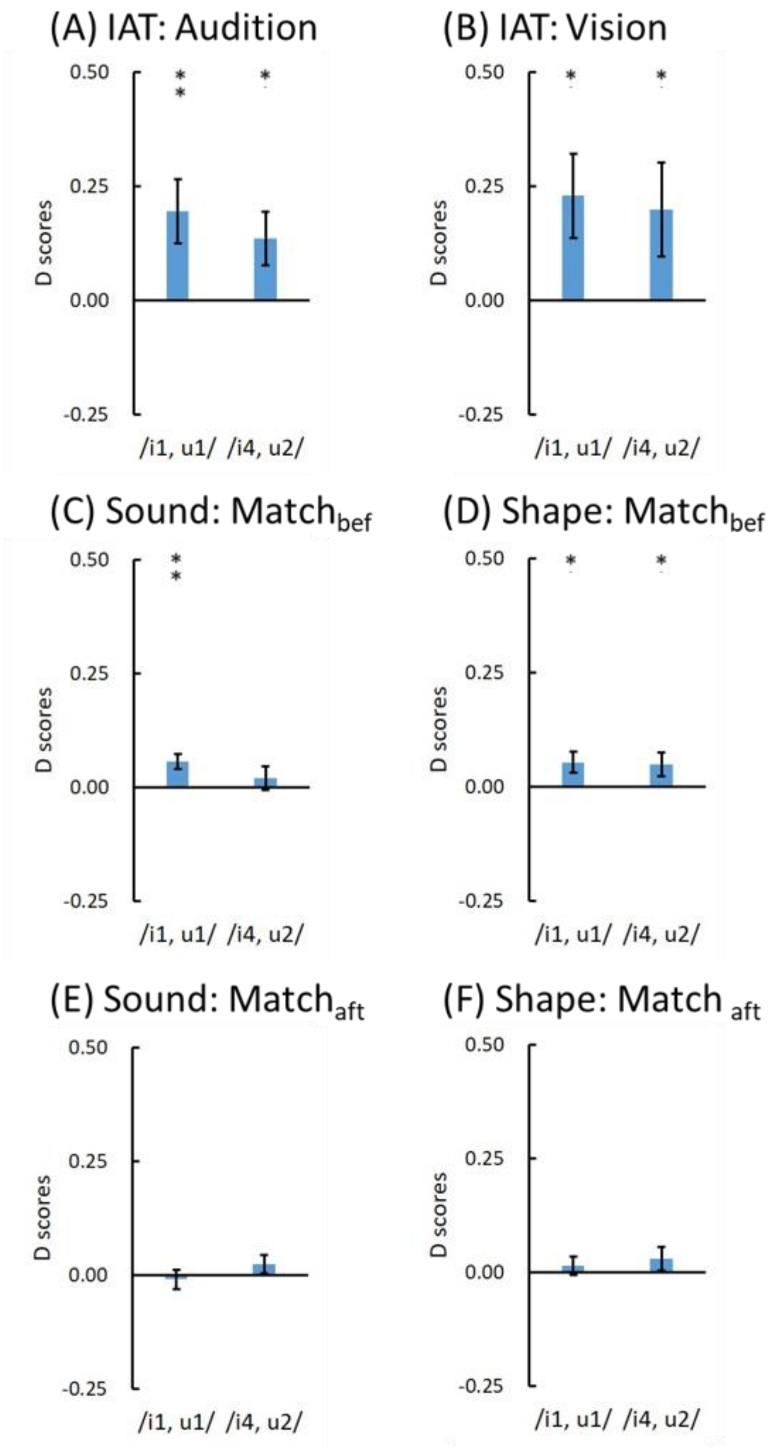

Figure S2. Mean d-scores in Experiments 1 to 3. The error bars represent the standard error of the mean. The asterisks indicate statistical difference from zero (* denotes p < .05, ** denotes p < .01)
